# Novel insights into PARPs in gene expression: regulation of RNA metabolism

**DOI:** 10.1007/s00018-019-03120-6

**Published:** 2019-05-04

**Authors:** Yueshuang Ke, Jing Zhang, Xueping Lv, Xianlu Zeng, Xueqing Ba

**Affiliations:** 1 The Key Laboratory of Molecular Epigenetics of the Ministry of Education, Institute of Genetics and Cytology, School of Life Science, Northeast Normal University, Changchun, 130024 Jilin China; 2 College of Life and Environmental Science, Wenzhou University, Wenzhou, 325035 China

**Keywords:** Poly ADP-ribosylation, PARP1, Gene expression, Transcription, RNA metabolism, RNA-binding proteins, Inflammation

## Abstract

Poly(ADP-ribosyl)ation (PARylation) is an important post-translational modification in which an ADP-ribose group is transferred to the target protein by poly(ADP-riboses) polymerases (PARPs). Since the discovery of poly-ADP-ribose (PAR) 50 years ago, its roles in cellular processes have been extensively explored. Although research initially focused on the functions of PAR and PARPs in DNA damage detection and repair, our understanding of the roles of PARPs in various nuclear and cytoplasmic processes, particularly in gene expression, has increased significantly. In this review, we discuss the current advances in understanding the roles of PARylation with a particular emphasis in gene expression through RNA biogenesis and processing. In addition to updating PARP’s significance in transcriptional regulation, we specifically focus on how PARPs and PARylation affect gene expression, especially inflammation-related genes, at the post-transcriptional levels by modulating RNA processing and degrading. Increasing evidence suggests that PARP inhibition is a promising treatment for inflammation-related diseases besides conventional chemotherapy for cancer.

## Introduction

The regulation of eukaryotic gene expression can be achieved at multiple levels, of which, RNA metabolism, including RNA synthesis, processing, and degrading, is the main level. While the mechanisms of transcriptional regulation have been extensively and intensively investigated, increasing amounts of data indicate that expression is also tightly regulated at the post-transcriptional level. Because modulations in the stability of mRNAs provide rapid and flexible controls, inflammation-related genes (such as cytokines, chemokines), growth factors, and proto-oncogenes are more dependent on post-transcriptional regulation [1, 2]. Pre-mRNA must undergo alternative splicing, polyadenylation, cytoplasmic translocation, and translation to transfer genetic information into the protein form. RNA-binding proteins (RBPs) play a central role during RNA transactions by acting as *trans* factors, and the dysregulation of RBP expression or function can cause a variety of human diseases [3–5]; however, little is known about the regulation of RBP activities in RNA metabolism. Recently, some RBPs that are post-translationally modified by poly(ADP-ribosyl)ation (PARylation) to regulate RNA processing, including splicing, polyadenylation, and mRNA turnover, have been identified [4, 6]. A new perspective of poly(ADP-ribose) polymerase (PARP) biology involving mRNA metabolism at the post-transcriptional level is emerging. In this review, along with updating the current knowledge on the roles of PARP in transcriptional regulation, we summarized the evidence showing the roles of PARPs and PARylation in other aspects of RNA metabolisms.

## Poly ADP-ribosylation and the PARP superfamily

**Fig. 1:**
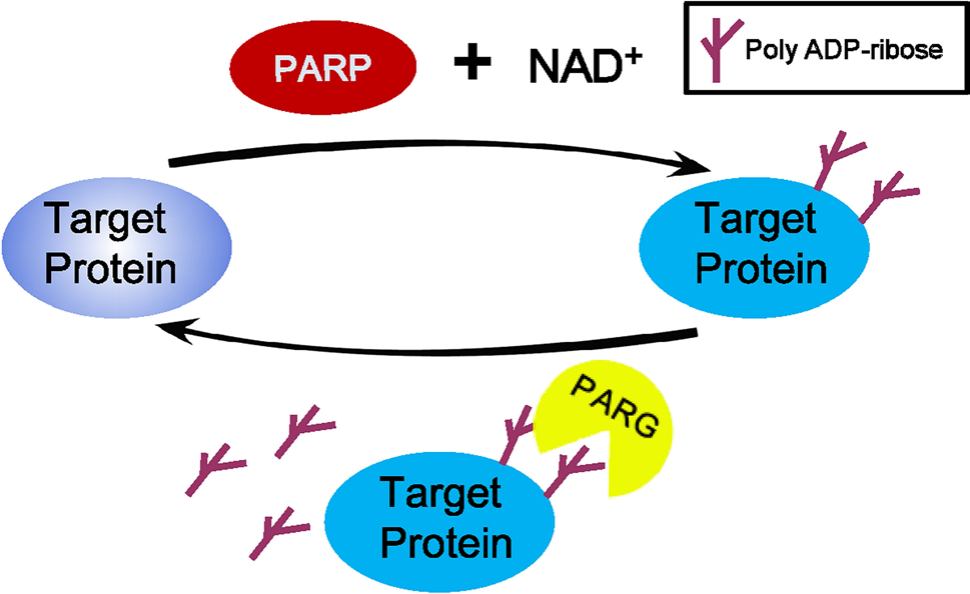
Poly ADP-ribosylation turnover. Poly (ADP-ribose) polymerase (PARP) utilizes NAD^+^ to create poly ADP-ribose, which then is attached to target proteins. In turn, the poly (ADP-ribose) glycohydrolase (PARG) removes poly ADP-ribose from target proteins, achieving the turnover of poly ADP-ribosylation

Using NAD^+^ as a substrate, PARPs catalyze the transfer of a negatively charged ADP-ribose polymer subunit to a target protein [7], and this post-translational modification is defined as PARylation (Fig. 1). Members of the PARP family are localized in a variety of cellular compartments, including the nuclei, cytoplasm and mitochondria [8]. Several PARP members (PARP1, 2, and 5) are currently considered to be poly-ADP-ribosyl transferases, while other PARP members (PARP 3, 4, 6–8, and 10–16) have been temporarily reclassified as mono-ADP-ribosyl transferases. Two members of this family (PARP9 and 13) appear to lack any enzymatic activity [9] (Table 1), and five PARPs (PARPs 7, 10, and 12–14) contain well-characterized RNA-binding domains, which are defined as RBPs [10]. The PARylation target proteins are either mono ADP-ribosylated or modified with 2–500 ADP-ribose units that profoundly affect the localization or function of the target protein [7]. PARylation is a reversible process, and the covalently attached PAR can be hydrolyzed to free PAR by PAR glycohydrolase (PARG) (Fig. 1). In addition to covalently PARylating proteins at specific amino acid positions, reading PAR signals by PAR-binding proteins constitutes a major aspect of PAR biology [11]. Besides the four distinct classical protein modules, PAR-binding motif (PBM), PAR-binding zinc finger (PBZ), WWE domains and Macro domains, well-characterized PAR reader modules are also newly reported, such as breast cancer 1 C-terminal, RNA recognition motif (RRM), serine/arginine-rich (SR) and lysine- and arginine-rich (KR) domains [9, 11, 12]. Free PAR acts as a death messenger, causing cells to undergo AIF-mediated cell death (parthanatos) [13–15], representing another major biofunction of PARylation that is beyond the scope of this review. The balance between the activation levels of PARP and PARG determines cell fate, by influencing both the level of energetic substrates (NAD^+^ and ATP) and amount of PAR, which has been investigated in recent studies [16–20].

**Table 1:** List of poly (ADP-ribose) polymerase (PARP) family members

PARP family member	Alternate name	Predicted size (kDa)	Subcelluar location	Motifs and domains	Enzymatic activity
PARP1	ARTD1	116	Nucleus	Zinc figers, WGR, BRCT	Poly
PARP2	ARTD2	63	Nucleus	WGR	Poly
PARP3	ARTD3	59	Nucleus	WGR	Mono
PARP4	ARTD4	190	Cytosol	BRCT	Mono
PARP5a	ARTD5, Tankyrase-1	146	Nucleus, Cytosol	Ankyrin repeat	Poly
PARP5b	ARTD6, Tankyrase-2	128	Nucleus, Cytosol	Ankyrin repeat	Poly
PARP6	ARTD17, tiPARPRMl	35	ND		Mono
PARP7	ARTD14	72	ND	Zinc figers, WWE	Mono
PARP8	ARTD16	94	ND		Mono
PARP9	ARTD9, BAL1	94	Nucleus, Cytosol	Macrodomain	
PARP10	ARTD10	113	Nucleus, Cytosol		Mono
PARP11	ARTD11	36	ND	WWE	Mono
PARP12	ARTD12, ZC3HDC1	77	Cytosol (stress granules)	Zinc figers, WWE	Mono
PARP13	ARTD13, ZC3HAV1, ZAP1	99	Cytosol (stress granules)	Zinc figers, WWE	
PARP14	ARTD8, BAL2, COAST6	198	Cytosol (stress granules)	Macrodomain, WWE	Mono
PARP15	ARTD7, BAL3	49	Cytosol (stress granules)	Macrodomain	Mono
PARP16	ARTD15	69	ND		Mono

*ARTD* ADP-ribosyl transferase, *ZC3HAV1* zinc-finger CCCH-type antiviral protein 1, *ZAP1* zinc-finger antiviral protein 1, *COAST6* collaborator of signal transducer and activator of transcription 6, *BAL* B-aggressive lymphoma protein, *ND* not determined, *Poly* poly-ADP-ribosyl transferases, *Mono* mono-ADP-ribosyltransferases

**Fig. 2:**
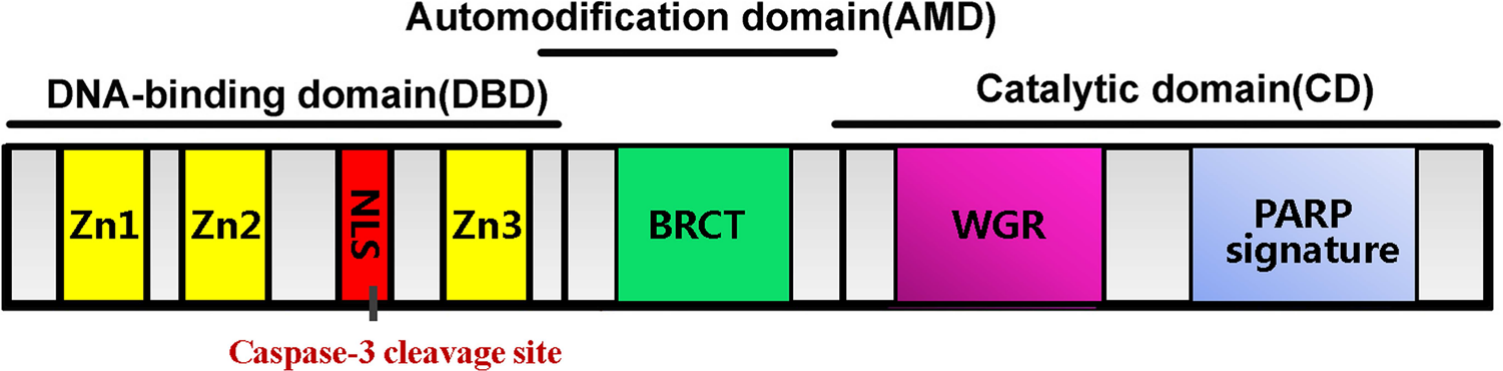
Structural and functional organization of PARP1. PARP1 contains the following structural and functional domains: (1) a DNA-binding domain (amino acids 1–372), containing three zinc finger motifs (Zn1, Zn2, and Zn3) and a nuclear localization signal (NLS); (2) an automodification domain (amino acids 372–524), containing a BRCA1 C-terminus (BRCT) motif; and (3) a catalytic domain (amino acids 525–1014), containing the WGR (Trp-Gly-Arg) motif and the highly conserved PARP signature motif

PARP1, the best studied and understood member of the PARP family, is a 116-kDa protein and can be divided into three functional domains. The N-terminal 46-kDa DNA-binding domain contains three zinc-binding domains (Zn1, Zn2, and Zn3) and a nuclear localization sequence. The first two zinc fingers appear to play different roles in the recognition of DNA breaks and enzyme activation, and they are both required to stimulate the activation of PARP1 in response to DNA single-strand breaks, but only the first zinc finger is required for the activation of PARP1 by double-strand DNA breaks [21]. A nuclear localization signal and a caspase-3 cleavage site within the DNA-binding domain are located between the Zn2 and Zn3 fingers. The central auto-modification domain contains a large number of glutamate and aspartate residues, which is consistent with the primary site of PARP1 self-modification. The C-terminal catalytic domain (54 kDa), which is the most conserved domain across the PARP family, consists of a WGR motif and the ‘‘PARP signature’’. The WGR motif is defined by a conserved Trp, Gly, and Arg residue-rich domain, and the ‘‘PARP signature’’ sequence is required for the catalysis of PAR synthesis [22] (Fig. 2). As the most abundant and ubiquitous member of the PARP family, PARP1 accounts for nearly 90% of cellular PAR formation after genotoxic stress [23]. In the absence of DNA damage, the constitutive level of PAR is very low, and the PAR chain is relatively short and typically presents as an oligomer of a few ADP-ribose units [24]. However, in response to DNA damage, the synthesis of long and branched PAR chains increases from 10- to 500-fold [25]. A large amount of data indicate that PARP1 can respond to different types and levels of stress. The levels of PARP1 activity and PAR synthesis increase as the strength of the stress stimulus increases, resulting in different cellular outcomes. The interaction with DNA breaks organizes PARP1 domains into a collapsed conformation, which can explain the strong activation of PARP1 [7], and, consequently, PARP1 itself is usually the primary target of PARP1-mediated PARylation. The excessive activation of PARP1 may cause the depletion of NAD^+^ and then ATP depletion, ultimately resulting in necrotic cell death [26]. Mild or moderate stress leads to DNA repair responses, which help maintain genomic stability or transcriptional regulation, such as regulating genes in response to inflammatory stimuli [27–29]. Under conditions in which abundant DNA breaks are lacking, post-translational modifications, such as phosphorylation, acetylation and methylation, are alternative mechanisms for the regulation of PARP1 activity [26, 27].

## PARP1 is involved in RNA synthesis/transcription

Over the past two decades, a growing amount of literature has revealed an important role for PARP1 in the regulation of gene expression. As an important nuclear protein, the altering of chromatin structure by modifying histones and acting as a component of enhancer/promoter-binding complexes are the two major mechanisms of PARP1 that affect transcription [30–32].

### PARP1 as a modulator of chromatin

A large number of reviews have comprehensively summarized that PARP1 can automodify itself or PARylate histones, thereby, altering the chromatin structure [30–33]. The linker histone H1 has been identified as the major acceptor of PARylation [32, 34]. PARP1 and histone H1 compete for binding to target gene promoters, which contributes to the dynamic regulation of gene expression [35, 36]. In addition, PARP1 can maintain an open chromatin formation by preventing the demethylation of H3K4me3. The PARylation of histone demethylase KDM5B inhibits its binding with chromatin, which promotes the loading of RNA polymerase II (RNA pol II) at the promoters of positively regulated target genes, allowing PARP1 to participate in transcriptional elongation [37]. As an intracellular sensor, PARP1 can be activated by various developmental signals and environmental cues, but how PARP1 is rapidly and specifically targeted to the physiologically correct gene loci is unknown. Recently, Hau and colleagues demonstrated that the transcriptional factors pre-B cell leukemia homeobox 1 (PBX1) and myeloid-like eco-integration site (MEIS) cooperate to induce chromatin opening by recruiting PARP1 when neural progenitor cells begin to differentiate into neurons [38]. Before differentiation, PBX1 is bound to the H1-compacted promoter/enhancer of the neuron-specific gene doublecortin (*Dcx*), essentially priming the gene for activation. Once differentiation is induced, MEIS associates with PBX1, and rapidly and specifically recruits PARP1 to the Dcx promoter/enhancer. This recruitment is associated with PARylation and the dismissal of histone H1, thereby facilitating *Dcx* gene expression [38]. The study suggested a novel mechanism and explained how PARP1 selectively targets promoters of downstream genes.

PARP1 is involved in the regulation of genomic DNA in the three-dimensional space. A chromatin insulator-binding protein CCCTC-binding factor (CTCF) has been shown to be PARylated by PARP1. For example, in *Drosophila*, PARylation of CTCF-interacting protein CP190 promotes its interaction with insulator DNA-binding proteins, suggesting that PARylation regulates the ability of insulators to mediate contacts between distant sites in the genome [39, 40]. Zhao et al. documented that PARP1 and CTCF not only mediate chromatin fiber interactions, but also trigger oscillations in the recruitment of circadian loci to the lamina, causing the silencing of these loci by the acquisition of repressive H3K9me2 and transcriptional attenuation [41]. More recently, studies showed that PARP1 cooperates with CTCF to regulate Epstein–Barr virus latency. PARP1 and CTCF colocalize at specific sites throughout the viral genome, and CTCF is PARylated by PARP1 to maintain the open chromatin landscape and transcription in type III latency [42]. Importantly, the inhibition of PARP1 activity by the PARP inhibitor olaparib abrogates the recruitment of CTCF to the C promoter, which is the key control point in distinguishing type III latency, resulting in decreased type III latency-related transcription [42, 43]. Data collectively suggested that PARP1 activity may be integral to the function of insulators in organizing the three-dimensional architecture of the genome to regulate gene expression.

On the other hand, free PAR generated by PARP1 has been shown to serve as a dynamic source of ATP, which is required for the activity of ATP-dependent chromatin-remodeling enzymes during cellular signaling [17]. Free PAR could be further broken down by pyrophosphatase NUDIX5 to produce ATP in the presence of pyrophosphate. The locally generated ATP participates in chromatin remodeling for the activation of transcriptional programs in response to hormone-dependent signaling [17].

### PARP1 as a transcriptional coregulator

In addition to modulating transcription through alterations in chromatin structure, PARP1 also regulates the activities of enhancers and promoters either positively or negatively [32, 44]. Nuclear factor kappa (NF-κB) exists as homo- or heterodimer that is made up of two subunits of RelA/p65, RelB, c-Rel, p50 and p52 [45]. NF-κB can be activated by PARP1 through various pathways. PARP1 may interact with histone acetyltransferases p300 and CREB-binding protein to activate NF-κB. Inflammatory stimulation triggers p300 and the CREB-binding protein’s acetylation of PARP1, enhancing PARP1’s interactions with p50, which would ultimately lead to NF-κB activation [46, 47]. Moreover, the enzymatic activity of PARP1 is important for NF-κB activation, and the PARylation of the p50/p65 dimer or automodification of PARP1 appears to be critical for their binding to DNA and subsequent transcriptional activation. For example, in lipopolysaccharide (LPS)-treated smooth muscle cells, PARP1 could PARylate p65 and increase the binding of p65 with CRM1, thus inducing p65’s nuclear retention and transcriptional function [48]. Additionally, in LPS-stimulated murine macrophage cells, the activated PARP1 PARylates the transcription factor NF-κB and promotes inflammatory genes’ (such as *Il*-*1β* and *Il*-*18*) mRNA expression [27].

In response to DNA damage, activated PARP1 can influence the function and location of the p53 transcription factor. During DNA damage, super-activated PARP1 induces the PARylation of p53 [49]. The E255, D256, and E268 sites of p53 are critical for its PARylation, and the PARylation of p53 specifically blocks p53’s interactions with the nuclear export factor CRM1, forcing p53’s nuclear localization and favoring the activation of p53-dependent genes [49]. In addition to covalent PARylation, p53 also undergoes a noncovalent high-affinity interaction with PAR [50]. A new study showed that p53 noncovalently bound to auto-PARylated PARP1 through its C-terminal domain, which placed p53 in spatial proximity with the catalytic center of PARP1, resulting in a covalent PARylation of p53 by PARP1 [18]. Thus, noncovalent binding by PAR and the covalent PARylation of p53 are co-dependent, which affects p53-dependent DNA-binding properties and transcriptional functions.

In addition, C/EBPβ, the key pro-adipogenic transcription factor, is PARylated by PARP1 on its K133, E135, and E139 amino acids, which are present in a conserved regulatory domain. The PARylation of C/EBPβ inhibits its DNA binding and transcriptional activities, which influences early events in the differentiation of adipocyte precursors [51].

## The function of PARP1 in RNA processing

New discoveries have demonstrated that many RNA-binding proteins (RBPs) are modified by PARylation, thereby regulating gene expression at the post-transcriptional level [4, 6]. These studies have revealed new aspects of the PARPs’ involvement in mRNA metabolism.

### Splicing

**Fig. 3:**
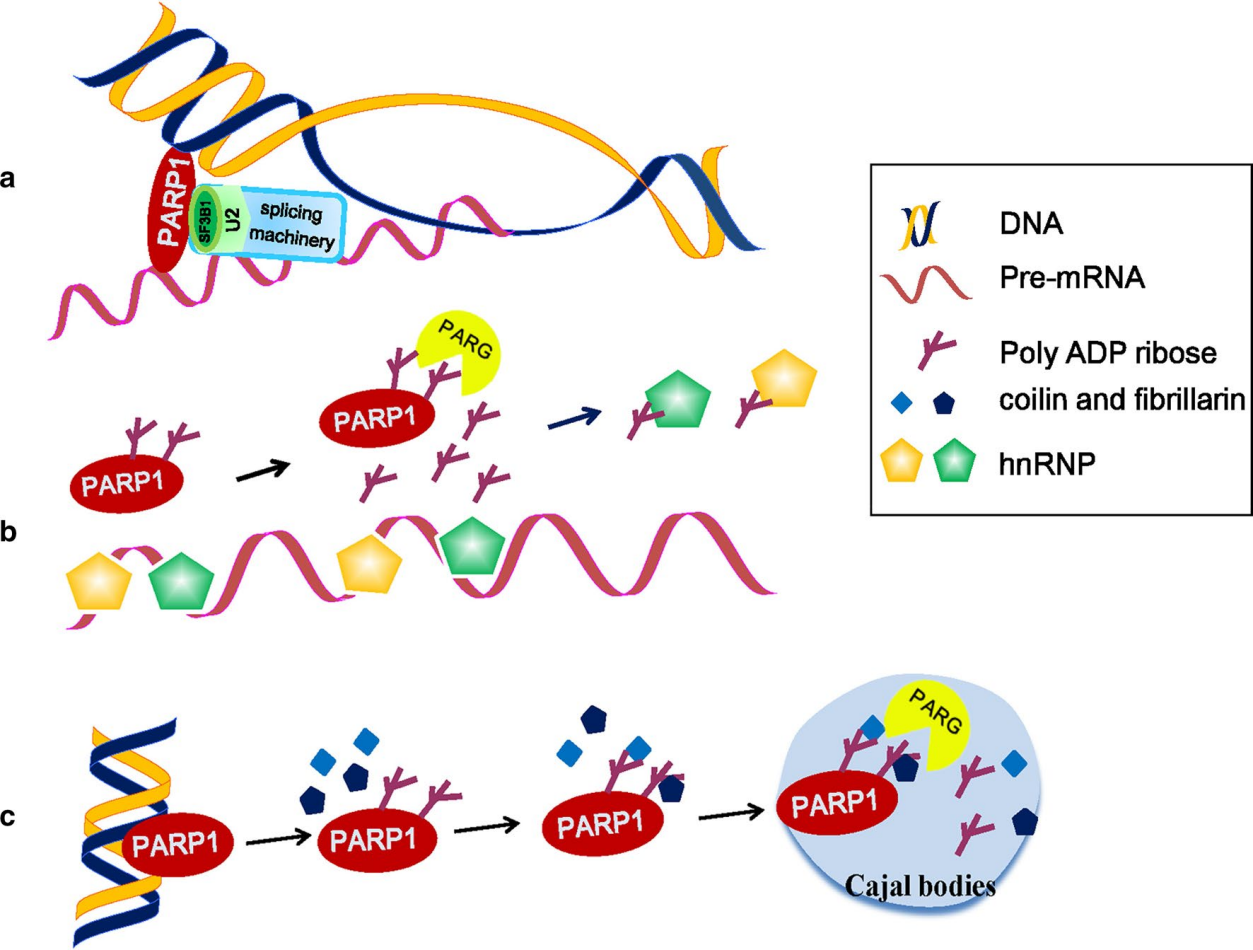
The function of PARP1 in RNA splicing. **a** Model of PARP1 in mediating alternative splicing. PARP1 binds to specific nucleosomes at exons and to the pre-mRNA. It then recruits the SF 3B subunit 1 (SF3B1), a component of the U2 snRNPs. U2 binds to the branch point recognized by the splicing machinery, allowing PARP1 to influence the splicing process [53]. **b** Model of PARP1 in the regulation of hnRNP functions. PAR binding to hnRNPs causes the dissociation of hnRNPs from RNA, which enhances or inhibits intron splicing [4, 59, 61]. **c** Model of protein delivery to Cajal bodies (CBs) by PARP1. Upon activation, autoPARylated PARP1 interacts with coilin and fibrillarin, and then the complex, consisting of auto-modified PARP1 and proteins, migrates into the CBs. In CBs, the complex is disassembled as a result of poly ADP ribose cleavage, and the released proteins are recycled [67]

Alternative splicing events play important roles in many cellular processes, including tissue-, cell type- and developmental stage-specific gene expression patterns in eukaryotes [52]. Alternative splicing is regulated by the binding of trans-acting factors to their target sites on pre-mRNA. These trans-acting factors promote or reduce the usage of a particular splice site [53]. In humans, almost 95% of multi-exonic genes are alternatively spliced [54, 55]. The “co-transcriptional splicing hypothesis” suggests that factors affecting chromatin structure and stability also regulate alternative splicing [56]. Intriguingly, a recent study documented that PARP1 acts as an adapter molecule bridging chromatin and nascent mRNA, and as a recruiter of splicing factors (SFs). PARP1 binds to nucleosomes at exon/intron boundaries corresponding to specific splice sites. Depletion of PARP1 or inhibition of its PARylation activity results in changes in the alternative splicing of a specific subset of genes [53]. PARP1 binds to specific nucleosomes (such as H3K4me3) at exons and to the pre-mRNA. It then recruits the SF 3B subunit 1 (SF3B1), a component of the U2 snRNPs. Knockdown of PARP1 impaired the association of SF3B1 to nucleosomes, whereas inhibition of PARylation had no significant effect [53]. U2 binds to the branch point recognized by the splicing machinery, allowing PARP1 to influence exon recognition and the splicing process [53] (Fig. 3a). PARP1 has been proved to be a novel mRNA-binding protein that preferentially binds RNAs containing GC-rich regions [57]. In the absence of the Zn1 and Zn2 domains, PARP1 switches from a DNA binder to an RNA binder, indicating that the Zn1 and Zn2 domains of PARP1 bind to chromatin and that PARP1 still has the ability to bind to mRNA through another domain [57]. A most recent follow-up study reported that PARP1 influences alternative splicing decisions through the regulation of RNAPII elongation. Genome-wide analyses confirmed that PARP1 influences the changes in RNAPII elongation by either reducing or increasing the rate of RNAPII elongation depending on the chromatin context, delineating PARP1’s role in RNA biogenesis and processing [58].

Additionally, PARP1 also plays a role in splicing by altering the RNA-binding dynamics of heterogeneous nuclear ribonucleoproteins (hnRNPs). In 2003, a proteomic approach demonstrated that hnRNPs can establish a strong noncovalent link with PAR. Specifically, the conserved domain located between RRM1 and RRM2 of hnRNPA1 is the PAR-binding motif [59]. HnRNPs, as a group of RBPs, in addition to the function in splicing, also mediate multiple steps of RNA processing, such as mRNA export, localization, translation, and stability [60]. To date, 11 human hnRNP proteins have been identified as PAR readers. In Drosophila, hnRNPs can either promote or inhibit splicing by binding with exonic and intronic splicing enhancers or silencers, and PAR binding to hnRNPs causes the dissociation of hnRNPs from RNA, which influences intron splicing [4, 61] (Fig. 3b). Alternative splicing factor/splicing factor 2 (ASF/SF2), a prototypical serine–arginine-rich (SR) protein, is involved in splicing regulation. PAR may bind to the splicing factor ASF/SF2 through either the RRM1 or SR-rich C-terminal domain, and it inhibits phosphorylation by DNA topoisomerase I in HeLa nuclear extracts [62]. DNA topoisomerase I has dual roles in transcription, controlling DNA supercoiling or acting as a specific kinase for the SR protein family of splicing factors [63]. Because ASF/SF2 phosphorylation can promote splicing [64], PAR binding to SR proteins may also regulate alternative splicing by modulating the phosphorylation of SR proteins.

Cajal bodies (CBs) are nuclear organelles in proliferative cells, which contain small nuclear ribonucleoproteins, small nucleolar RNPs, RNA pol II transcription factors and nuclear proteins [65]. CBs are involved in many RNA metabolic processes, such as transcription and pre-mRNA splicing [66]. Under stress conditions, autoPARylated PARP1 gains the ability to bind to a number of nuclear proteins. At this time, the automatically modified PARP1 acts as a shuttle protein to deliver the protein components to the CB to regulate its assembly and disassembly and to further regulate transcription and splicing [67]. For example, under physiological conditions, unmodified PARP1 binds to chromatin and accumulates in the nucleolus. Upon activation, autoPARylated PARP1 interacts with coilin and fibrillarin, two key protein components of CBs, in a PAR-dependent manner and increases the migration of the two proteins from chromatin into CBs. In CBs, the complex is disassembled by PARG cleavage, and coilin and fibrillarin proteins are released [67] (Fig. 3c). These findings indicate that PARP1 and PAR affect transcription and splicing by regulation CB biogenesis and dynamics.

### Polyadenylation

**Fig. 4:**
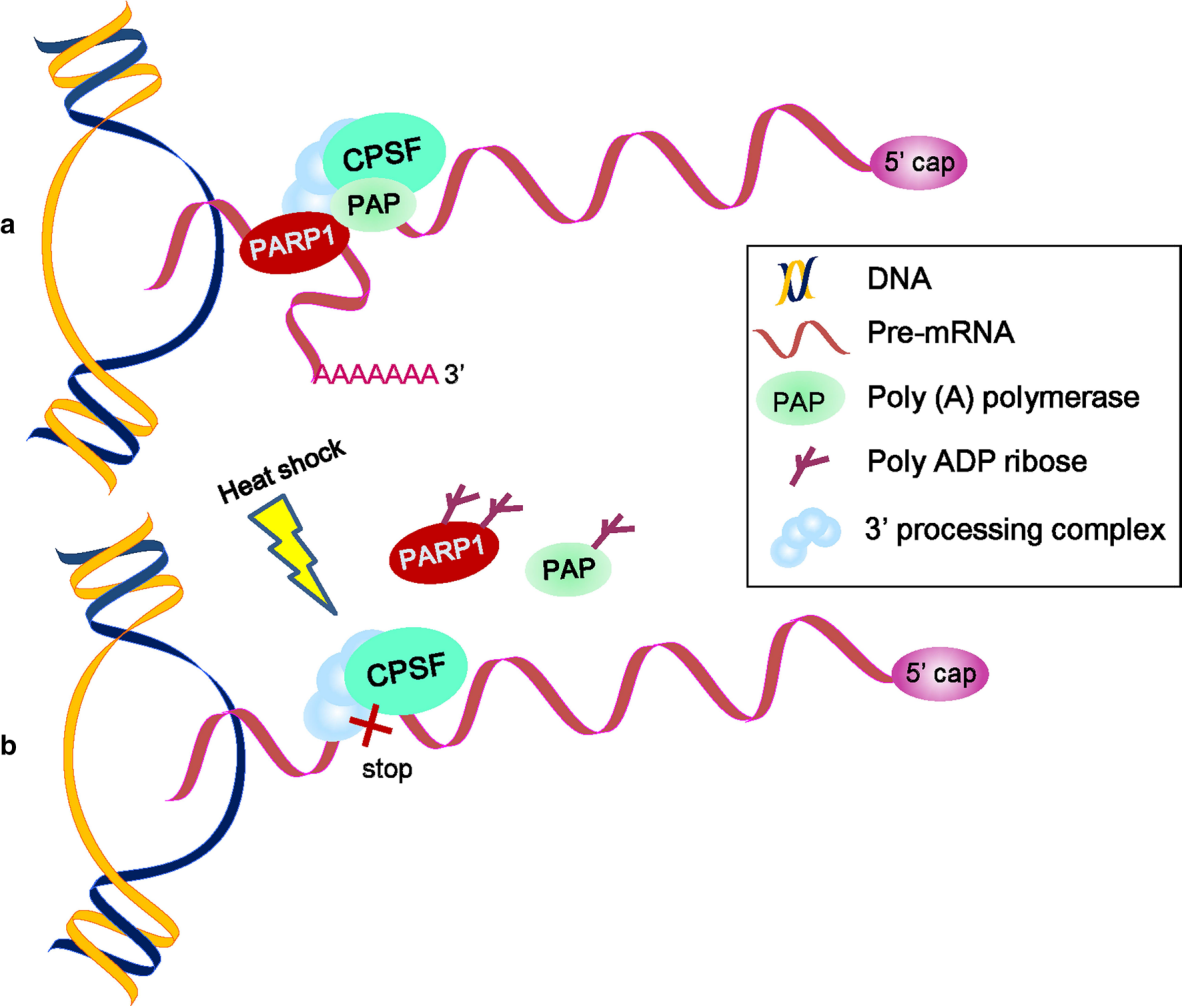
The function of PARP in pre-mRNA polyadenylation under heat-shock conditions [70]. **a** Under normal conditions, polyadenylation specificity factor (CPSF) and poly(A) polymerase (PAP) together with the 3′-processing complex bind pre-mRNAs. Pre-mRNAs’ 3′cleavage and polyadenylation occur normally. **b** The activated PARP1 PARylates PAP under heat-shock conditions, which causes the dissociation of PAP and PARP1 from RNA, leading to the inhibition of pre-mRNA polyadenylation and the repression of mRNA maturation

Remarkably, almost all human pre-mRNAs possess multiple cleavage sites and polyadenylation signals in their 3′-untranslated region (3′-UTR) [68]. The generation of mature mRNA 3′ ends primarily consists of two reactions, endonucleolytic cleavage catalyzed by polyadenylation specificity factor and the synthesis of a poly(A) tail onto the 5′-cleaved product by poly(A) polymerase (PAP). The human pre-mRNA 3′-processing complex contains approximately 85 proteins, and PARP1 has been identified as a related factor, implying a role for PARP1 in processing complex assembly and function [69, 70] (Fig. 4a). PARP1 binds and PARylates PAP in vitro, which in turn prevents PAP from associating with most mRNA transcripts, inhibiting polyadenylation and mature mRNA synthesis under heat-shock conditions [70] (Fig. 4b). Surprisingly, the γ-irradiation (γ-IR) and oxidative stress are unable to redirect PARP1 activity toward PAP and inhibit polyadenylation [70]. Because PARP1’s activation during heat shock may be directed by some unknown molecular mechanism, PARylated PAP influences the mRNA maturation process only under heat-shock conditions. Polyadenylation contributes to many aspects of mRNA metabolism, including mRNA export to the cytoplasm, mRNA stability, and the translation efficiency [71]. During heat shock, the polyadenylate-ome subjected to regulation by PARylated PAP may be necessary for us to further understand the crucial role of PARP1 in mRNA processing.

### Nuclear export

**Fig. 5:**
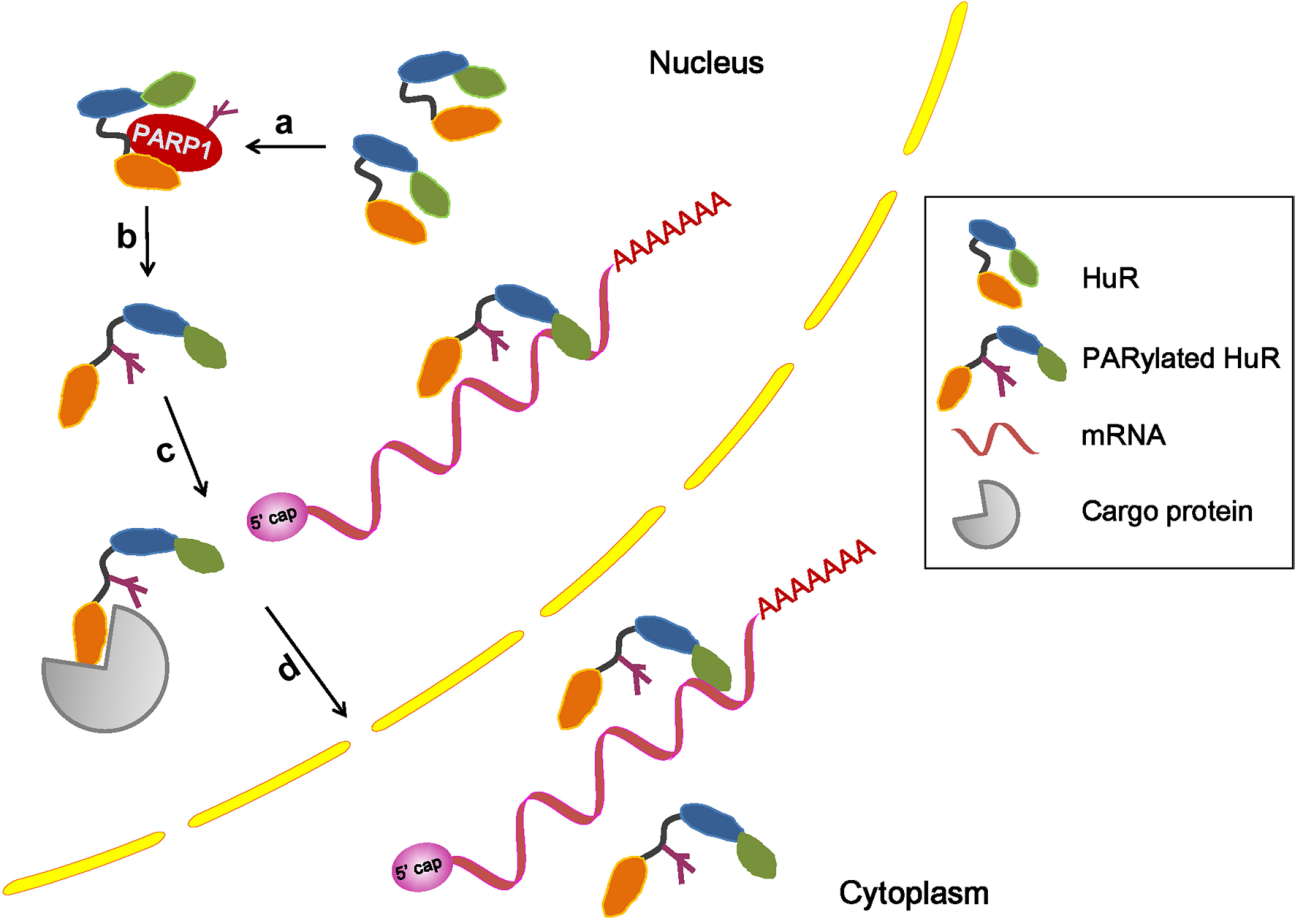
Model in which PARP1 PARylates HuR and influences its location and function [6]. **a** The activated PARP1 binds and PARylates the HuR protein; **b** the PARylation of HuR alters protein properties; **c** the exposure of the HuR nuclear export sequence offers more opportunities for it to be recognized by carrier proteins; and **d** the accumulation of HuR in cytoplasm leads to the functional activation of HuR, allowing it to bind target mRNAs

Eukaryotic gene expression requires the export of protein-coding mRNAs and non-coding RNA molecules from the nucleus to the cytoplasm. The major classes of cellular RNAs are exported as RNA–protein complexes, which involves RBPs as well as receptor proteins that interact with the nuclear pore complexes [72]. Although most mRNAs use transcription/export, transcription/export -2, and nuclear RNA export factor 1 receptors to transit through nuclear pore complexes, a subset of mRNAs use chromosome maintenance region 1 (CRM1) as the receptor. As a key nuclear export receptor, CRM1 does not directly bind RNA or protein. Instead, CRM1 associates with adaptor proteins by recognizing a leucine-rich nuclear export signal [73]. Those mRNA containing AU-rich elements (AREs) in their 3′-UTRs belong to the early response genes [2], and the ARE-binding protein HuR, as an adapter protein, links RNA to CRM1 using the ligands pp32 and APRIL [74]. The HuR–CRM1 pathway mediates the export of some ARE-containing mRNAs, such as c-fos and CD83 [75, 76]. A recent study identified CRM1 together with HuR as nuclear exports that mediate the exportation of long non-coding RNA (lncRNA) RMRP [77]. RMRP is encoded by a nuclear DNA but has key functions in mitochondria, which are important for mitochondrial DNA replication and RNA processing [78, 79]. Furthermore, we found that in cells exposed to LPS, PARP1 binds to HuR and increases HuR’s PARylation at the D226 site. PARP1 inhibition or D226 mutation diminishes the enhanced nucleocytoplasmic translocation of HuR [6]. While the hypomethylation at R217 and dephosphorylation at S221 have been attributed to the impaired interaction of HuR with CRM1 and its subsequent mislocalization [80], PARP1’s involvement in CRM1-mediated RNA nuclear export is plausible, although the study did not address the requirement of HuR’s PARylation for its interaction with CRM1, either directly or indirectly (Fig. 5). Interestingly, it has been reported that p65 PARylation decreased its interaction with CRM1 in vitro. The pharmacologic inhibition of PARP1 increased the NF-κB–CRM1 interaction in LPS-treated smooth muscle cells, suggesting that p65 PARylation may be a critical determinant for the interaction of NF-κB with CRM1, NF-κB nuclear retention and gene expression upon TLR4 stimulation [48]. These studies implied that PARP1 has different effects on the CRM1-mediated nucleocytoplasmic shuttling of the PARylated cargo proteins.

## A novel function of PARP1 in regulation of mRNA stability

The amount of protein generated from any given mRNA depends not only on the rate of mRNA translation, but also on the rates of mRNA synthesis and decay. Thus, mRNA stability provides an additional crucial layer of regulation at the post-transcriptional stage of gene expression. Genetic deletions of PARP1 or pharmacological blockades of PARPs activity in mice result in defective inflammatory immune responses [29]. However, the role of PARP1 in the regulation of inflammatory gene expression at the post-transcriptional level, and especially its effects on mRNA stability, is still less studied. Interferon-γ-inducible protein-10 (IP-10), a member of the CXC family of chemotactic factors, is a potent chemoattractant for T- and natural killer cells [81, 82]. In 2010, Galbis-Martínez et al. showed that PARP1’s depletion did not influence the activity of the IP-10 promoter, but the expression of *IP*-*10* mRNA induced by interferon (IFN)-γ is diminished in *Parp1*^−/−^ mouse embryonic fibroblasts, suggesting that PARP1 regulates the *IP*-*10* mRNA level by increasing mRNA stability, although the study did not dissect the precise molecular mechanism of how PARP1 influences the mRNA level of IP-10 [83].

The regulation of mRNA stability involves both *cis* elements that are harbored in mRNA and the cognate *trans* factors. Among them, ARE and ARE-binding proteins have attracted the most attention. Up to 8%–10% of the genes in the human genome contain at least one putative ARE in their 3′-UTR [84]. AREs are found in the tightly regulated mRNAs that encode proto-oncogene proteins (e.g., c-Fos and c-Myc) and inflammatory mediators (e.g., CXCL2 and IL-1β). Most ARE-binding proteins function as negative regulators by decreasing mRNA stability or translation, e.g., tristetraprolin, AU-rich element RNA-binding protein 1 and TIA-1-related protein [2], while HuR is one of the few that stabilizes ARE-containing mRNAs.

HuR is a member of the embryonic lethal abnormal vision family. Unlike other members (HuB, HuC, and HuD) of the family, which have tissue-specific expressions, HuR is ubiquitously expressed in almost all types of cells [2, 85]. HuR is localized predominately in the nucleus and shuttled into the cytoplasm using a shuttling sequence HNS under various stress conditions [86]. HuR may compete with degradation proteins and prevent them from transporting the mRNAs to the decay structure, the exosome [87]. The functional regulation of HuR is achieved through protein modifications, such as phosphorylation, methylation and ubiquitination [19, 88–92]. Recently, it was reported that inflammatory stimuli can induce PARP1 interactions with HuR and PARylates it at the D226 site, which is located in the HNS domain. It was documented that the PARylation could not only enhance the nuclear–cytoplasmic shuttling of HuR, but also increase the association of HuR with target mRNAs. Unlike other studies that usually investigated the binding of HuR with mRNA in cytoplasm, the increased formation of protein–mRNA complexes upon HuR PARylation was detectable both in the nucleus and cytoplasm, which may mean PARylation not only enhances shuttling of HuR but also accelerates nuclear cytoplasmic delivery of HuR’s target. PCR array analyses of inflammatory cytokines and chemokines showed that the PARP1–HuR signal influences the stability of a set of ARE-containing mRNAs, such as *Cxcl1, Cxcl13*, and *Il*-*1β* [6]. The mechanism of how PARP1–HuR interaction regulates mRNA stability is illustrated as follows: (a) the activated PARP1 binds with and PARylates HuR at the HNS domain; (b) the PARylation alters the conformational properties of the HuR protein, which likely exposes the nuclear export sequence for translocation and the RRM for binding with mRNAs; (c) the exposure of nuclear export sequence increases the opportunity of HuR (itself or complexed with mRNAs) to be recognized by export proteins, such as CRM1 or transportin-2; and (d) the accumulation of HuR in the cytoplasm leads to the functional execution of HuR (Fig. 5). Recently, Chand et al. showed that, in pancreatic ductal adenocarcinomas cells, under DNA-damaging conditions, PARP1 binds with HuR and increases the shuttling of HuR to the cytoplasm, where HuR stabilizes PARG mRNA and promotes protein expression, enhancing DNA repair [93]. Thus, there appears to be a new aspect of PARP1 biology in gene expression regulation at the post-transcriptional level by stabilizing target mRNAs.

## Other PARPs in RNA regulation

Although much of the focus has been on PARP1, studies over the past decade have begun to reveal the physiological and pathological roles of the other PARP family members, with many new and exciting functions being identified [31, 94–96]. Below, we highlight some of the key results regarding cytoplasmic PARPs in RNA regulation.

**Fig. 6:**
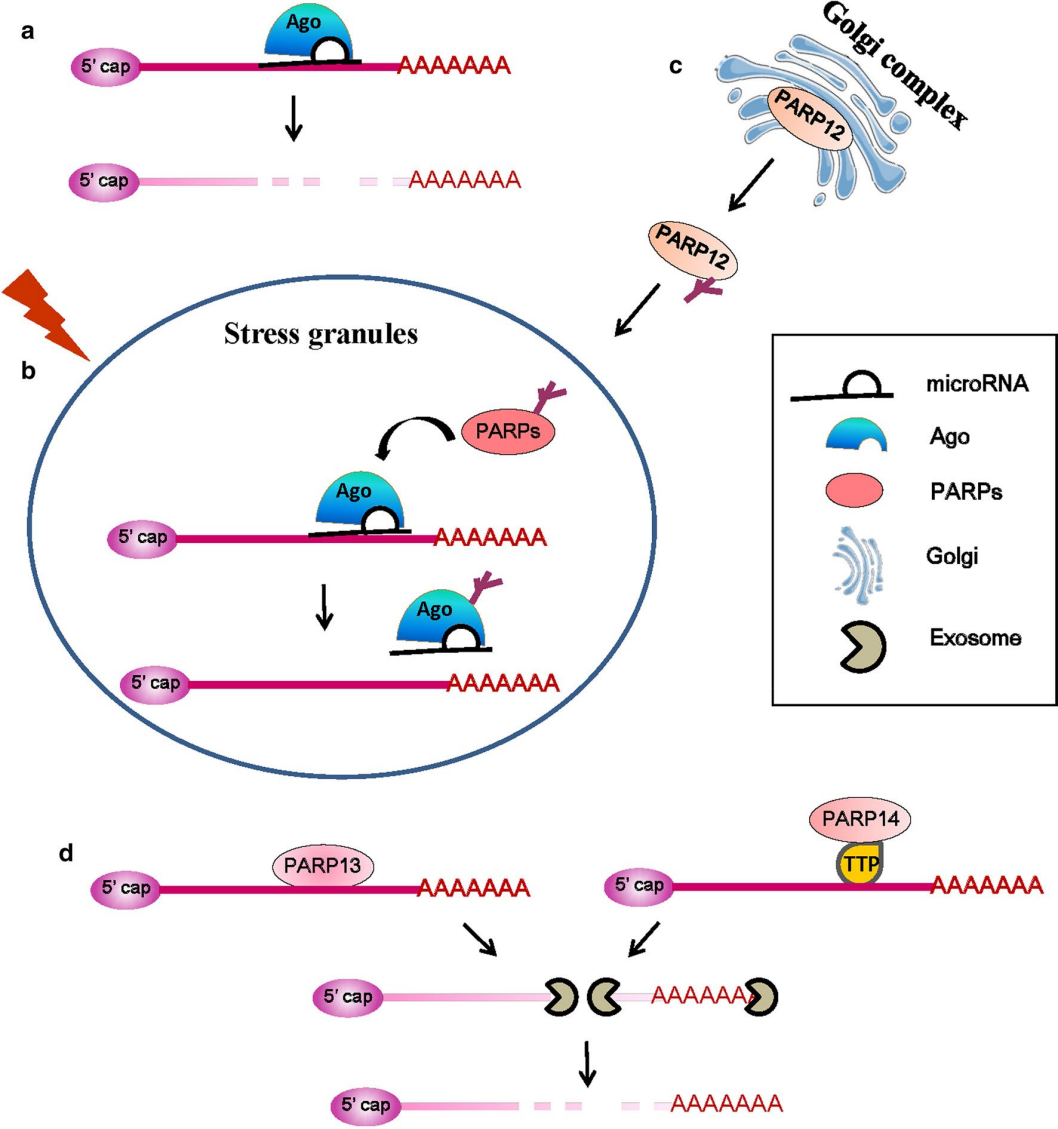
PARP family members involved in RNA regulation. **a** MicroRNAs and Ago bind to target mRNAs, which leads to the decay of the target mRNAs. **b** The activated PARP family member PARylates the Ago protein in stress granules (SG), which decreases it’s binding with target mRNAs and reduces its silencing activity under stress conditions [98]. **c** The activation of PARP1 and PAR release increase PARP12’s translocation from the Golgi complex to SG [100]. **d** PARP13 and PARP14 promote the degradation of specific transcripts by targeting them to the cellular RNA decay machinery [102, 105]

mRNA and microRNA (miRNA) form dense aggregated structures called stress granules to stop translation when cells are under stress conditions [97]. Stress granules are cytoplasmic, non-membranous structures that form within minutes of exposure to stress and dissolve within a few hours of stress recovery. Six members of the PARP family, including PARP5a, 12, 13.1, 13.2, 14, and 15, together with two forms of PARG (PARG99 and PARG102) in the cytosol participate in the miRNA-related activities in the stress granules. MiRNAs bind to target mRNAs, which leads to the translational repression and decay of the target mRNAs, and this process requires the Ago protein (Fig. 6a). The PARylation of Ago decreases its binding with target mRNAs and reduces its silencing activity (Fig. 6b) [98].

PARP12, a Golgi-localized single ADP-ribosyltransferase of the PARP family, functions in cell survival and anti-viral responses [99]. The activation of PARP1 and PAR release from the nucleus lead to PARP12 translocation from the Golgi complex to stress granules (Fig. 6c), and the zinc finger and WWE domains of PARP12 are necessary for its PAR binding and translocation [100]. PAR binding with PARP12 contributes to shutting down translation and to the cross talk between members of the PARP family involved in gene expression at the post-transcriptional level.

PARP13, also known as ZC3HAV1 and zinc-finger antiviral protein (ZAP), is an RBP. PARP13 binds RNAs of viral origin during infection and then recruits RNA-decay factors, such as exosome complexes to degrade RNA [101]. PARP13 also regulates miRNA through Ago inhibition by targeting Ago proteins for PARylation [98]. The global repression of miRNA silencing results in the up-regulation of pro-inflammatory cytokines and common miRNA targets, and helps mount the antiviral response. In addition to its antiviral functions, PARP13 also binds to cellular RNA. A major target of PARP13 regulation is TNF-related apoptosis-inducing ligand receptor 4 (TRAILR4) mRNA, which encodes a member of the TRAIL receptor family. PARP13 destabilizes TRAILR4 mRNA post-transcriptionally, but has no effect on the mRNA levels of other TRAIL receptors [102]. PARP13 binds to the 3′-UTR of TRAILR4 through its CCCH domain and promotes TRAILR4 mRNA degradation via the RNA exosome complex [101] (Fig. 6d left). Thus, by targeting TRAILR4 mRNA, PARP13 acts as a pro-apoptotic factor and may increase the cellular response to TRAIL in transformed cells [102].

PARP14, a member of the PARP family, is a pro-survival protein that has been reported as being involved in protecting lymphocytes from apoptosis [103, 104]. Additionally, a recent study showed that PARP14 is involved in mRNA stability regulation [105]. PARP14 interacts with tristetraprolin, one of the dominant mRNA-destabilizing factors [68], and forms a complex that binds to the 3′-UTR of tissue factor mRNA, thereby promoting its degradation [105] (Fig. 6d, right).

## Involvement of PARPs in inflammation

Inflammation plays critical roles in host defense responses and damage repair, and it is also a common underlying cause of many diseases and is recognized as a hallmark of many cancers [106, 107]. Over the past years, a large number of studies have addressed the involvement of PARPs in the progression of different pathological processes, such as lung inflammatory disorders, cardiovascular disorders, breast cancer, and diabetes [27, 47, 108–116]. Here, we focus on the implications of PARPs in the process of inflammation.

Pro-inflammatory factors, such as cytokines and chemokines, are key mediators of inflammation and host defenses, and their regulation is largely dependent on related transcription factors. As mentioned above, many transcription factors that are involved in the inflammatory process, such as activator protein-1 (AP-1), p53 and NF-κB, have been reported to be regulated by PARP1 [18, 48, 108–114, 117, 118]. The PARylation of the transcription factors will affect their intracellular localization, stability, or ability to bind with the target gene promoter, which in turn affects the expression of pro-inflammatory factors [117–121]. For example, PARP1 increases the production of pro-inflammatory cytokines, such as TNFα, IL-5, and Cxcl2, by activating the NF-κB pathway, which plays important roles in the pathogenesis of asthma, acute lung injury, and chronic obstructive pulmonary disease [27, 108, 122]. Kiefmann et al. showed that PARP1 contributes to acute lung injury via the up-regulation of iNOS through the activation of AP-1, but not NF-κB during endotoxemia [123]. In addition, the mRNA stability of a series of pro-inflammatory chemokines (*Cxcl1*, *Cxcl2*, *Cxcl13*, and *Il*-*1β*) is influenced by PARP1–HuR signals (see above) [6], which also suggests a potential strategy to treat diseases closely associated with mRNA stability. Furthermore, HuR can bind PARG mRNA under PARP inhibitor stress, and the inhibition of HuR combined with PARG mRNA helps to improve the effectiveness of PARP inhibitory therapy in pancreatic cancer cells [93]. The RNA-binding proteins hnRNPs, which noncovalently bind with PAR, are involved in many neurodegenerative diseases, including fragile X syndrome, Alzheimer’s disease, and amyotrophic lateral sclerosis [4]. Although the study did not define PARP1’s function, PAR binding to hnRNPs caused the dissociation of hnRNPs from RNA, suggesting a reduction in hnRNP’s noncovalent bonds with PAR by a PARP inhibitor may improve treatments for neurodegenerative diseases [4, 61, 124] (Table 2).

**Table 2:** Studies on the roles of PARPs involved in inflammation

PARPs	Factors	Target genes	RNA metabolism	Functions	Pathological process/model	References
PARP1	NF-kB	TNFa, INFy, IL6, MMP...	Transcription	PARP1 fuels NF-KB-driven inflammation gene expression	Asthma, breast cancer, diabetes...	[48, 108–114]
PARP1	AP-1	iNOS	Transcription	PARP1 contributes to acute lung injury via up-regulation of iNOS through activation of AP-1	Acute lung injury	[123]
PARP1	NFAT	IL-2	Transcription	PARPl positively regulate NFAT-dependent cytokine gene transcription	Acute inflammation	[117]
PARP1	STAT-6	IL-5	Transcription	PARPl affects IL-5 expression through regulation of STAT-6 nrotein inteeritv	Asthma	[122]
PARP1	Snail	E-cadherin	Transcription	PARPl inhibits E-cadherin promoter activity by upregulating Snail protein stability	Malignant melanoma	[120]
PARP1	HMGB1		Transcription	PARPl increases the pro-inflammatory mediator HMGB1 accumulation in the cytosol	Necrosis	[119]
PARP1	KLF8	CyclinDl	Transcription	PARPl increases cancer related transcriptional factor KLF8 nuclear location and transcriptional activity	Breast cancer	[121]
PARP1	HuR	PARG	mRNA stability	Inhibition of HuR combines with PARG mRNA helps to improve PARP inhibition therapy in pancreatic cancer cells	Pancreatic ductal adenocarcinoma	[93]
PARP1	HuR	Cxcl2	mRNA stability	PARPl promotes pro-inflammation gene expression by modulating the RNA-binding protein HuR	Acute inflammation	[6]
PARP1	HnRNP	FMR1	mRNA splicing	PAR binding to hnRNPs causes the dissociation of hnRNPs from RNA	Neurodegenerative diseases	[4, 60, 61, 124]
PARP1	p38	IP-10	mRNA stability	PARPl regulates inflammatory mRNA stability via modulating p38 MAPK signalling _A_ pathway	Acute inflammation	[83]
PARP2				The deletion of PARP2 reduces spinal inflammation	Neuroinflammation	[126]
PARP10	NF-kB	IL-8	Transcription	PARPl0 represses NF-KB-dependent inflammation related gene and protein expression	Chromic inflammation	[127]
PARP12	NF-kB	IL-8	Transcription	PARPl2 activate the NF-kB signal and promotes IL-8 secretion in response to an extracellular ligand	Microbial infections	[99]
PARP13		TRAILR4	mRNA stability	PARPl3 binds to 3′UTR of TRAILR4 mRNA, and leads to its degradation via the RNA exosome complex	Apoptosis	[102]
PARP14	STAT6	Gata3	Transcription	PARPl4 modulates the binding of STAT6 to the Gata3 promoter	Allergic airway disease	[128]
PARP14	PKM2			PARP 14 promotes the Warburg effect by inhibiting JNK1 -dependent PKM2 phosphorylation	Hepatocellular carcinoma	[129]

*STAT-6* signal transducer and activator of transcription-6, *EMT* epithelial–mesenchymal transition, *HMGB1* high mobility group box-1 protein, *KLF8* kruppel-like factor 8, *PKM2* pyruvate kinase M2 isoform

Studies on other members of the PARP family in the inflammatory process have also emerged. The closest homolog of PARP1 is PARP2, which is able to PARylate itself or other target proteins [125]. PARP2 has also been reported to regulate neuroinflammatory responses. *PARP2*^−*/*−^ mice showed an attenuated neuronal inflammation and reduced spinal inflammatory cell infiltration and demyelination [126]. Furthermore, the PARP members localized in the cytoplasm participate in the regulation of inflammatory gene expression. In response to the stimulation of cytokine IL-1β or TNFα, PARP10 represses the activation of NF-κB and downstream the expression of target genes and proteins (i.e., IL-8) in HeLa and U2OS cells [127], whereas the overexpression of PARP12 activates NF-κB signaling and promotes IL-8 secretion in response to an extracellular ligand [99]. The RNA-binding protein PARP13 is another PARP family member. In addition to its role in targeting viral RNA degradation, its activity has also been reported to prevent cancer [101]. By inhibiting the expression of the pro-survival receptor TRAILR4, PARP13 helps to sensitize cells to TRAIL-mediated apoptosis, thereby limiting cancer cell survival [102]. In fact, multiple cancerous tumors exhibit a low expression of PARP13 compared with normal tissues, suggesting that PARP13 is an important pro-inflammatory and pro-apoptotic agent [101]. Additionally, PARP14 is highly expressed in diverse solid tumors, PARP14-deficient animals show reduced lung pathology compared with control animals [128], and PARP14 promotes the Warburg effect by inhibiting JNK1-dependent PKM2 phosphorylation [129] (Table 2). Thus, PARP inhibition may extend to treatments of inflammation-related diseases, besides its conventional application in cancer therapy.

## Conclusions

**Fig. 7:**
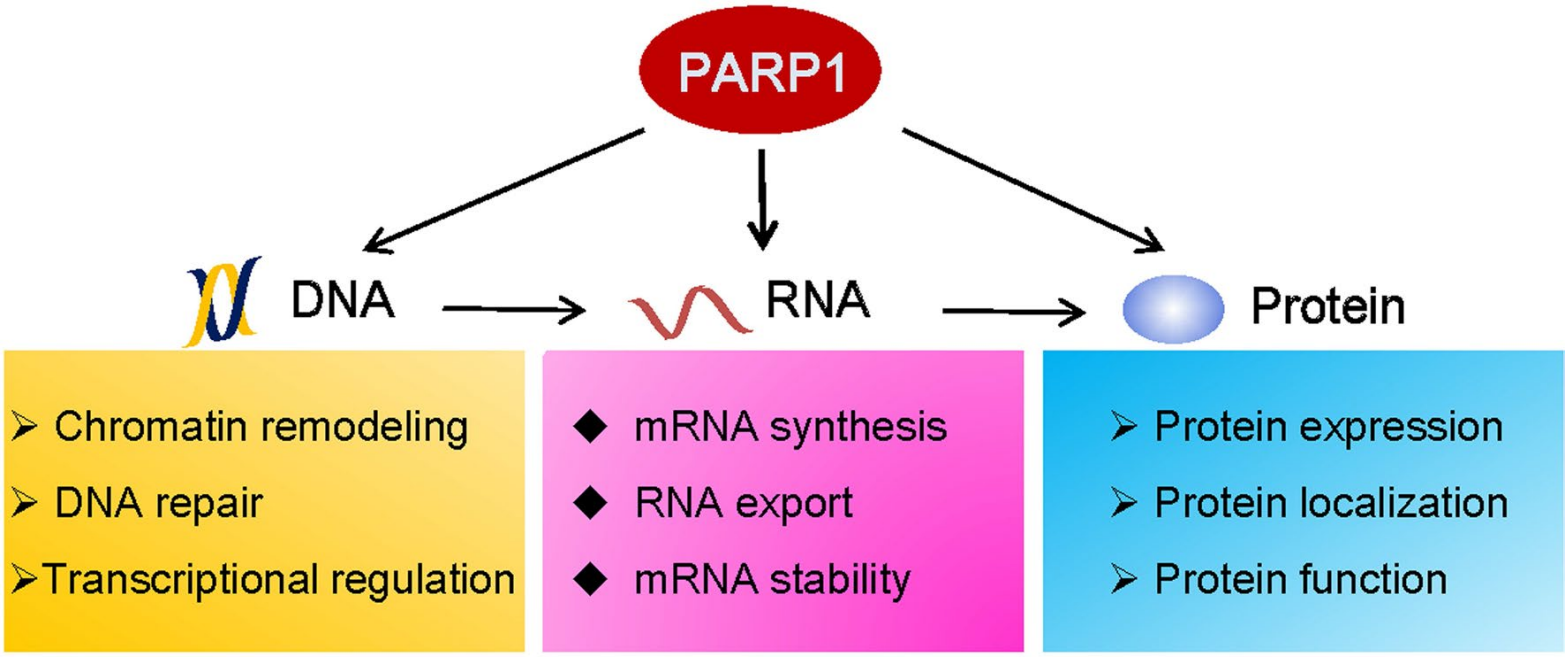
PARP1 modulates cellular stress responses through a series of regulatory processes that occur at the DNA, RNA, and protein levels

PARPs modulate the molecular biology and biochemistry of stress responses at multiple levels. In addition to the important roles of PARP1 in DNA damage detection and repair, its roles in RNA biology, gene regulation, and cytoplasmic functions have now emerged (Fig. 7). Although a large body of evidence has accumulated over the past decade, many questions still remain. Our understanding of the roles of PARPs in the regulation of gene expression, especially at the post-transcriptional level, is still in the early stage. PARPs, together with PARG, participate in many RNA metabolism-related processes, such as splicing, polyadenylation, mRNA maturation and translation by manipulating the functions and locations of RBPs. Recently, PARP1 was identified as a novel RBP. Although the range of mRNA types bound by PARP1 has not been explored in detail, it has been implied that PARP1 plays multiple and complex roles in RNA metabolism. In conclusion, the regulation of RNA processing by PARPs occurs at nearly all of the key steps. RNA regulation is an exciting area of PARP biology, but much work remains to be performed.

## Abbreviations

RBPs: RNA-binding proteins
PARylation: Poly(ADP-ribosyl)ation
3′-UTR: 3′-Untranslated region
PAR: Poly-ADP-ribose
PARP: Poly(ADP-ribose) polymerase
PARG: PAR glycohydrolase
PBX: Pre-B cell leukemia homeobox
MEIS: Myeloid-like eco-integration site
Dcx: Neuron-specific gene doublecortin
CTCF: CCCTC-binding factor
NF-κB: Nuclear factor kappa B
RNA: Pol II RNA polymerase II
ASF/SF2: Alternative splicing factor/splicing factor 2
SR protein: Serine–arginine-rich protein
HuR: Abnormal visual 1 (Elavl1)/human antigen R
RRM: RNA recognition motif
hnRNPs: Heterogeneous nuclear ribonucleoprotein
CBs: Cajal bodies
PAP: Poly(A) polymerase
CRM1: Chromosome maintenance region 1
AREs: AU-rich elements
TRAIL: TNF-related apoptosis-inducing ligand
AP-1: Activator protein-1

## Notes

Compliance with ethical standards
